# Nudge or not, university teachers have mixed feelings about online teaching

**DOI:** 10.1057/s41599-023-01691-1

**Published:** 2023-05-12

**Authors:** Sanchayan Banerjee, Beatriz Jambrina-Canseco, Benjamin Brundu-Gonzalez, Claire Gordon, Jenni Carr

**Affiliations:** 1 Institute for Environmental Studies, Vrije Universiteit Amsterdam, Amsterdam, The Netherlands; 2 London School of Economics and Political Science, London, UK

**Keywords:** Psychology, Education

## Abstract

We designed and administered an online survey experiment to 444 educators in a large social sciences university in the United Kingdom to evaluate their perceptions on the effectiveness of online teaching methods. We find that a nudge, designed to inform educators about the benefits of online teaching, does not improve the personal evaluations of educators in our sample (*n*_treat_ = 142, *n*_control_ = 142) about this new mode of teaching. Overall, most respondents in our sample report being comfortable with online teaching methods and think this form of teaching can continue to have some positive impact. Nonetheless, they do not favour any further online transition away from traditional modes of teaching. Online teaching is largely perceived by a majority of these educators to negatively affect student well-being and their overall university experience. We call for more experimental research in higher educational settings to evaluate the role of *edunudges* in improving the uptake of online teaching tools.

## Introduction

A few months after the outbreak of COVID-19, Witze (2020) reported *how universities will never be the same after the crisis*. Time seems to be proving her right. As the pandemic unfolded, the higher education sector was forced to make drastic changes, a process that is reshaping how universities continue to function. Online teaching, for instance, while initially thought of as a pandemic blip, is morphing into a permanent reality of public education (Kaufman and Diliberti, 2021). But this online transition comes with new pedagogical and cognitive costs for students and teachers alike (Pokhrel and Chhetri, 2021; Psotka, 2022). And while there is some early scholarly evidence on the learning experiences of students in online environments (Almendingen et al., 2021; Flores et al., 2022; Kuntz and Manokore, 2022; Orlov et al., 2021; Raaper et al., 2022; Sarvary et al., 2022; Warfvinge et al., 2022), teachers’ emotions and views about online teaching methods remain largely unexplored (Bartlett, 2021; Jones and Kessler, 2020). We contribute to fill this gap in the literature by designing and administering an online survey experiment to 444 educators of a large social sciences university in the United Kingdom to evaluate their experiences on the effectiveness of online teaching methods.

There is growing evidence that university teachers faced difficulties in adapting to online modes of teaching (Lee et al., 2022, Power et al., 2022), with many educators reporting low levels of professional satisfaction (Li and Yu, 2022). Notable differences in the digital proficiency and literacy of educators have been attributed to variations in demographic characteristics, such as their age and gender, and to the availability of training and support (Vergara-Rodríguez et al., 2022). While these challenges in adopting online teaching methods varied amongst educators, they nonetheless existed for almost all academics in university settings globally (Lee et al., 2022). However, whether such experiences represent the true pitfalls of an emerging online pedagogical toolkit remains under debate. It is likely that these bad experiences have been jointly shaped by the pandemic, the rushed transition it forced towards online teaching methods, and the socio-emotional effects it had on educators more generally. This conjecture is further supported by prior scholarly evidence that points towards the many benefits of online education at large (Dyment et al., 2020; Huang, 1997; Mayadas et al., 2009; Muir et al., 2019; Stone, 2021; Stone et al., 2019; Summers et al., 2023). Therefore, as online teaching methods become more prominent in higher educational institutions, it is important to disentangle the role of the pandemic in shaping educators’ teaching experiences. Our study is timely in this regard, as we are able to study the immediate feelings of educators (in a large social sciences U.K. university) who employed these online teaching tools during peak periods of the pandemic outbreak in the United Kingdom.

Finally, it is expected that if educators feel overwhelmed and face heightened emotions, such as higher levels of work stress and burnouts (Kaufman and Diliberti, 2021; Ma et al., 2021; Mosleh et al., 2022; Ozamiz-Etxebarria et al., 2021), it might disproportionately reduce their teaching efficacy, which can then spillover negatively on student learning experiences and well-being (Oberle et al., 2020). Therefore, it is important to improve teaching experiences by supporting educators, and instructing them on the benefits that a transition to online teaching methods can have more widely (Dodo-Balu, 2017, 2022). Prior evidence of using such behavioural techniques to nudge educational outcomes is sparse, and their use to “reshape key aspects of faculty work and decision-making” is almost non-existent (O’Meara et al., 2022, p. 277). We also contribute here, as we designed and administered a behavioural nudge to educators in our sample that reminded them of the benefits of online education. To the best of our knowledge, our study is the first to design and then test randomly an information nudge to improve educators’ perceptions of the effectiveness of online teaching methods during the pandemic. This nudge was delivered as part of the mentioned online survey experiment which was then administered to all levels of the university’s teaching community between April and May 2021.

We use a mixed-methods approach to analyse educators’ views and perceptions of the effectiveness of online teaching methods. While existing studies evaluating similar sentiments have been mostly descriptive in nature and primarily relied on qualitative analyses (see Ma et al., 2021), our experimental findings are unique and add to a growing base of robust quantitative evidence that points towards a low acceptability of online teaching methods during the pandemic. Specifically, we find that faculty who responded to the survey report being comfortable with online teaching methods generally. They also believe this form of teaching can continue to have some positive impact on education. Nonetheless, they do not favour any further online transition away from traditional modes of teaching. Online teaching is largely perceived by educators in our sample to negatively affect student well-being and their overall university experience. Further, our nudge, informing educators in the sample (*n*_treat_ = 142, *n*_control_ = 142) of the benefits of online teaching does not improve their personal evaluations about this new mode of teaching. Our findings point towards mixed feelings about online teaching methods, from educators who have used such tools in a large social sciences university in the United Kingdom during the pandemic.

The remaining paper is organised as follows. Section “Literature review” briefly reviews the literature on sentiments of educators in response to a transition to online teaching during the pandemic, and on the use of behavioural nudges to steer outcomes in higher education settings. Following this, the section “Research design and methods” describes our research design and methods. We summarise our findings in the section “Results” before concluding with the limitations of our study design and future research directions in the section “Conclusion”.

## Literature review

### Sentiments towards online teaching in university settings

The outbreak of the pandemic introduced socio-economic changes, ripples of which were felt globally across different sectors of the economy. One such change impacted the education sector, mainly in how education was imparted to students. Traditional in-person modes of teaching were quickly replaced by online modes of education, such as those to be delivered using internet-enabled platforms and devices, either synchronously using *Zoom*, *MS-Teams*, *Google Hangouts*, or asynchronously using pre-recorded audio-visual content. In most cases, this transition was completely unprecedented as governments imposed “hard” measures to contain the spread of the pandemic (see Banerjee et al., 2021). These large-scale changes had a direct impact on students and teachers within the education sector.

While this impact on students is well evidenced and documented (Almendingen et al., 2021; Flores et al., 2022; Kuntz and Manokore, 2022; Orlov et al., 2021; Raaper et al., 2022; Sarvary et al., 2022; Warfvinge et al., 2022), the same is not true for teachers, particularly those in university settings. So much so, that many scholars (see Bartlett, 2021; Kaufman and Diliberti, 2021) have voiced concerns about the teaching community being largely neglected and/or ignored. In a systematic review of 21 studies, for instance, Li and Yu (2022) show a general decline in levels of satisfaction among teachers. Further, there is also evidence of moderate to high levels of work stress and burnouts among university teachers (Kaufman and Diliberti, 2021; Ma et al., 2021; Mosleh et al., 2022; Ozamiz-Etxebarria et al., 2021). This can be explained by a lack of training, support, and teaching resources that were available to university teachers in assisting them with a transition to online modes of teaching. There is evidence for this in rich and poor countries alike, such as in Australia (Dodo-Balu, 2017, 2018, 2022), Bangladesh (Saha et al., 2022), China (Liu et al., 2022; Tsegay et al., 2022), Egypt (Elewa et al., 2022), Japan (Kita et al., 2022), Pakistan (Shahid et al., 2022; Yasmin, 2022), Sweden (Hietanen and Svedholm-Häkkinen, 2023), Vietnam (Nguyen et al., 2022), and the United States (Sessions et al., 2022).

We situate our study in this emerging pool of evidence about the sentiments of university teachers towards online teaching during the pandemic. In doing so, we present evidence using a sample of 444 educators based at a large social sciences institution in the United Kingdom. We chose this institution as our case study for three main reasons. First, being one of the largest social sciences institutions in the United Kingdom, this university presented us with a unique test bed of multidisciplinary courses that are offered to students as a degree option. These courses vary in their use of quantitative and qualitative methods of teaching and learning. We believe this is an important advantage, as we expected the transition to online education during the pandemic would have disparate effects on the teaching delivery and experience of educators based on the individual teaching needs of the subjects taught. Second, this university hosts one of the most internationally diverse communities of students and teachers globally, which therefore enabled us to access a diverse pool of respondents to include as subjects in our survey experiment (see Table 17). Third, this university, being one of the leading higher educational institutions in the country and globally, meant our experimental findings could become readily actionable and shared as a good practice among other higher educational institutions to improve educators’ experience of online education going forward^1^.

### Nudges in education

A nudge refers to “any aspect of the choice architecture that alters people’s behaviour in a predictable way without forbidding any options or significantly changing their economic incentives” (Thaler and Sunstein, 2009, p. 6). In other words, a nudge alters how choices are presented to people, so they can make better decisions. Although there is considerable debate on the definition of a nudge (see Banerjee and John, 2022), there is agreement that a nudge should not prevent people from making decisions by either banning choices or making them more expensive. For example, placing items at an eye-level, automatically defaulting people into a higher savings option from which they can opt-out, reordering items on a menu, or making information more salient are all examples of nudges. Conventional nudges take advantage of people’s biases, and engineer choices in ways that lead citizens to perform behaviours as intended by the “nudger” or choice architect (see Baldwin, 2015). Such behavioural nudges have been widely used to improve different behaviours (for a review see Egan, 2013). More recently, such behavioural nudges have been extended to improve educational outcomes and behaviours.

Implementations of *edunudges*—educational nudges (Decuypere and Hartong, 2023)—are limited (O’Meara et al., 2022). Many of the current applications of nudges in improving educational behaviours are reduced to theoretical expositions of why such interventions might be useful (see Brinkmann, 2017; Brown et al., 2022; Damgaard and Nielsen, 2018; Decuypere and Hartong, 2023; Lynch, 1997; O’Meara et al., 2022; Weijers et al., 2021). Where empirical evidence exists, such randomised evaluations are sparse and are limited to students and/or teachers influenced by text-based messages to improve educational outcomes (Hanno, 2023; Taylor et al., 2022). In other settings, personalised support is also available to nudge educators towards better learning outcomes (Azzolini et al., 2023; Hustus, 2021; Pugatch and Wilson, 2018).

We contribute to this emerging literature on edunudges by designing and testing randomly an information nudge that improves the salience of online education for educators. We discuss this nudge in more detail in the section “Experimental design”.

## Research design and methods

### Survey design

We designed and administered an online survey experiment to all levels of the university’s teaching community between April 1 and May 20, 2021. This survey was designed to elicit the online teaching experiences of faculty in the *Lent Term*—running from January until March of the 2020–2021 academic year. The survey was distributed via email three times in the April–May period, during which time we received 444 responses—representing ~31% of the academic community teaching in the 2020–2021 academic year. The distribution of the email worked as follows: the university’s educational enhancement centre sent an email with the survey link to all departmental managers, who then circulated it among all employed faculty members within each department.

We decided to limit our period of study to the Lent Term to try and isolate views and preferences about online teaching specifically. This was because of the following reason: similar to other educational institutions in the United Kingdom, during the Michaelmas term of the same academic year—from September to December 2020—the university had implemented a hybrid arrangement. In this hybrid arrangement, students could either be in the classroom or follow the in-person sessions over *Zoom*. This process was fraught with technical difficulties and led to many concerns being voiced against it by the teaching community^2^. In addition, hybrid teaching was not implemented evenly, with some educators teaching only in-person, others exclusively online, and yet others opting for the hybrid approach, depending on individual or departmental preferences. Thus, considering how different teaching experiences may have been for the same person over both terms, we decided to focus all questions on the Lent Term, where all teaching shifted to an online format and therefore only prompted respondents to recall their experiences during this time.

We designed the survey in consultation with members of the university’s educational enhancement centre^3^. The survey was divided into four main parts. In part 1, we collected background information on the teachers’ main educational roles and commitments in the 2020–2021 academic year. In part 2, we elicited teachers’ perceived notions of effectiveness of teaching, learning, and assessment in response to the online mode of teaching during the Lent Term. These survey questions were provided with a 5-point Likert scale, ranging from “Strongly Agree” to “Strongly Disagree”. In part 3, we asked teachers about their common tools and methods of teaching. They were also asked to self-report their pedagogical beliefs about a successful transition to online teaching using a battery of six questions. Finally, in this part, we asked teachers if they felt supported by (a) their department, (b) the university’s educational enhancement centre, and (c) the university in general in making this transition to online teaching. Every survey question in this part was provided with a similar 5-point Likert response scale, ranging from “Strongly Agree” to “Strongly Disagree”. In part 4 of the survey, we randomly delivered an information nudge to teachers and consequently evaluated their beliefs about the effectiveness of online teaching methods in response to this treatment. At the end of part 4, we asked all teachers to self-report standard demographic information, following which the survey ended. The survey is available in the Online Appendix.

Next, we describe the experimental design of our survey in more detail.

### Experimental design

We embedded an online experiment in the survey, aiming to test whether teachers could be nudged to consider online teaching more positively. We did this using a simple information nudge that increased salience on the general benefits of online education. Participating faculty members were randomised into a treatment and a control condition using *Qualtrics’* in-built randomiser function, such that only the educators in the treatment condition received the information nudge. Set up this way, we were able to evaluate the causal role of the educative information nudge in improving educators’ perceptions of online teaching methods.

In the treatment condition, the information nudge reminded teachers of the benefits of online education. Specifically, they were presented with the text outlined below:

The British Council, in a recent blog titled *Is online learning the future of education?* stated that, “the world wide web has helped make learning an enjoyable, multimedia experience. Although some argue that the remote nature of the web can isolate us, as it reduces the need for face-to-face contact, online learning is an inherently social experience based around online conversation. The integration of digital technology into education has had a profound impact, opening distribution globally and allowing flexible, on-demand, around-the-clock services for learners. It also connects us to vast stores of information.”

Our information nudge was framed in a neutral manner and was broad in its goals. We made this choice for two main reasons. First, there is substantial evidence that framing affects decision-making (for a review, see Grüne-Yanoff, 2016). Our nudge conveyed many of the benefits of online education such as attracting a diverse audience ("opening distribution globally and allowing flexible, on-demand, around-the-clock services for learners”), and the convenience and flexibility it offers ("it reduces the need for face-to-face contact”), while also highlighting its negatives ("some argue that the remote nature of the web can isolate us”) to avoid framing bias. Second, our nudge was designed to reduce the cognitive demand it could have on participants. As such, the nudge was pre-tested extensively with members of the university’s educational centre, so as to understand its fit to the context. It is worthwhile to consider that when the survey experiment was fielded there were active discussions around online education in the university—so it is likely that many (if not all) faculty members were already sufficiently informed about the specific pros and cons of online teaching. Finally, our choice for the British Council as the source of this information was based on the institution’s relevance and credibility to education in the UK.

Following this experimental intervention, we asked teachers about their preferences for online teaching and learning. In particular, all teachers were asked if they agreed (using a 5-point Likert scale) with the following 8 statements:

- Digital fatigue is hampering student learning (*Teacher Fatigue*).
- Digital fatigue is hampering my teaching delivery (*Student Fatigue*).
- I feel comfortable in continuing with online teaching methods (*Comfort with Online Teaching)*.
- The School should increasingly switch to online teaching methods (*Switch to Online Teaching*).
- Online teaching methods could continue to have a positive impact on teaching and learning (*Positive effects*).
- A move to online teaching will negatively impact student well-being (*Well-being*).
- Online teaching will negatively impact how students enjoy the [school] experience (*Negative University experience*).
- I want to continue with some online teaching options but do not want them to completely replace traditional modes of teaching [*Continue partially*)].

### Research methods

We used a mixed-methods approach to analyse the outcomes of this online survey experiment. In the section “Results”, we first report descriptive statistics summarising individual characteristics of educators in our sample. Then we report on their pedagogical attitudes and beliefs as collected in parts 2 and 3 of our survey. Specifically, we report teachers’ average levels of perceived effectiveness of teaching, learning, and assessment; their pedagogical beliefs about a successful transition to online teaching; and perceived levels of support from the department, university, and its educational enhancement centre.

Following this, we present the experimental findings from the online experiment in the survey. Here, we estimated the average treatment effect of the information nudge using linear regression models to evaluate if the experimental treatment produced any measurable difference across any of the eight different measures of educators’ preferences for online teaching and learning. We controlled for covariates selectively using Lasso (Bloniarz et al., 2016) and corrected for multiple hypotheses using standard Westfall-Young (Jones et al., 2019) and Romano-Wolf (Clarke et al., 2020) step-down *p*-values.

Besides this quantitative analysis, we also analysed the open-ended text questions in the survey qualitatively to provide contextual information relevant to the implementation of our experiment. For example, across parts 2–4, our survey included open-ended questions, where teachers could elaborate on their reasoning behind their answers and/or add any relevant comments. In total, we collected 965 comments on issues related to individual teaching experiences, changing beliefs around teaching, and details on the support provided to deliver online teaching. We undertook a thematic analysis of these responses to help interpret and give context to the experimental results (for more details, see section 5 in Online Appendix).

## Results

### Descriptive statistics

#### Sample characteristics

Our survey received 444 responses, representing a third of the university’s teaching community in the 2020–21 academic year. Just over half of these responses were from teachers employed on a contractual basis in the university, such as graduate teaching assistants, postdoctoral teaching fellows, and guest teachers. Of those who held a permanent position, such as assistant professors or equivalent and above, ≈80% were on a research and teaching career development track, whereas ≈13% were from a teaching (or education) only track. At least one teaching member of staff participated from all 27 departments in the university, with the highest rates of absolute participation from the departments of *Geography and Environment*, *Language Centre*, *Management*, *Mathematics*, and *Social Policy*. More than 90% of our educators had prior university teaching experience, with 11 years being the modal experience in the sample. More than two-fifths of the sample had a teaching load of 2 or more courses in the *Lent Term*. The educators in our sample had an almost even split of teaching either undergraduate or postgraduate degree programmes, with a-third teaching both. Less than 20% of the educators in our sample held a teaching qualification or equivalent. In terms of its demographic composition, ≈56% of the sample included female educators whereas ≈70% of it was over 35 years of age. A detailed list of these summary statistics is available in section 5 (see Tables 3–17 and Figures 2, 3) of the Online Appendix.

#### Attitudes and beliefs of educators

In this section, we describe the pedagogical attitudes and beliefs of educators in our sample. These attitudinal measures were collected prior to the online experiment embedded in the survey, and therefore represent pre-treatment beliefs that were unaffected by the information nudge. Table 1 shows these perceived notions of teaching effectiveness. We find that most of the university teaching community (69%) is satisfied with their overall teaching experience. In general, this sentiment appears to stem from student engagement with the staff’s teaching delivery (70% agree), with course contents (76%), from the attainment of course objectives (83%), and from how much students appeared to enjoy the course (82%).

**Table 1:** Responses to pre-treatment survey questions.

	Strongly agree	Somewhat agree	Neither a. nor d.	Somewhat disagree	Strongly disagree
Overall, I feel satisfied with my online teaching experience	25.9	43.3	12.5	14.1	4.3
I feel satisfied with the level of student engagement with my teaching delivery	26.6	44.3	11.2	13.4	4.6
I feel satisfied with the level of student engagement with their peers	11.5	30.2	22.0	25.3	11.2
Students have achieved the intended learning objectives for my course(s)	42.6	40.7	11.2	4.9	0.7
I feel satisfied with the level of student engagement with the course content(s)	30.8	45.9	11.5	9.8	2.0
Students will be able to reach their full potential with online learning	17.4	32.5	13.8	23.6	12.8
I can accurately gauge student understanding through online teaching	17.7	37.4	15.1	21.0	8.9
Online summative assessment methods truly reflect student learning	20.3	28.5	28.2	16.7	6.2
Students have enjoyed my course(s)	38.6	44.2	15.2	2.0	0.0
Students have enjoyed this adapted [School] experience	6.6	26.7	24.4	31.4	10.9
Students accept that [the School] has adapted effectively under difficult circumstances	28.4	48.8	17.2	4.3	1.3
I have successfully adapted my teaching delivery to suit online learning	35.3	47.2	10.8	5.6	1.1
I have spent more time preparing for online teaching than I would need to for in-person teaching	57.7	18.2	12.9	7.7	3.5
I have changed my expectations regarding student learning due to the pandemic	15.4	37.8	20.3	14.3	12.2
I have set more lenient marking criteria for summative marking assessments due to the pandemic	4.6	25.2	32.5	19.6	18.2
I believe that online teaching has fostered independent student learning	9.4	31.5	29.7	22.0	7.3
I believe that online teaching has fostered peer interaction and exchange of ideas	8.7	18.5	23.8	25.9	23.1
I feel supported and enabled by my department in delivering online teaching	38.6	36.1	16.1	6.0	3.2
I feel supported and enabled by the [educational enhancement centre] in delivering online teaching	20.7	37.2	29.5	8.1	4.6
I feel supported and enabled by the School in delivering online teaching	15.8	34.0	24.2	17.9	8.1

Next, we note that the evidence on the perception of student learning is mixed. Only half of the teaching community agreed with the statement “[s]tudents will be able to reach their full potential with online learning”, and 36% outright disagreed. Further, only half of the community believed they can accurately gauge student understanding while teaching online. In addition, 53% of respondents reported having changed their expectations about student learning. Evidence regarding the perception of student engagement is also mixed: only 41% of the teaching community reported being satisfied with the level of student interaction among themselves and only a third of the educators thought that students had enjoyed this adapted university experience. Nonetheless, a large majority (77%) of educators thought that their students believed that the university had adapted effectively under a set of difficult circumstances. We find these perceptions were shaped mainly from the end-of-teaching surveys (such as TQARO surveys) and/or informal feedback obtained from students (such as during office hours) – for details, see Table A.17. in the Online Appendix.

Additionally, the survey itself reflects a change in the perceptions about the assessments employed to evaluate student learning: 30% of the teaching community reported, at least to some extent, to have set more lenient marking criteria. Nonetheless, about half of the teachers surveyed believed that online summative assessment methods truly reflected student learning, with only a quarter stating the opposite.

The survey finds evidence of an increased and successful effort by the university teaching community to adapt their delivery to online teaching. 75% of educators reported spending more time preparing for online classes than they would have needed for in-person teaching, regardless of the level of experience of the respondent. Some of these efforts were directed towards learning to employ online and recording tools that were in fact new for many of the educators. In this regard, teachers report having introduced several web-based technologies to facilitate online teaching. *Zoom* breakout rooms and polls, online handouts, audio-visual materials, and *Moodle* forums are featured as the most used tools in online classes. But significant time also went into adapting the delivery and activities used in discussion-based seminars to formats that would enhance student participation in an online setting. Overall, these efforts seem to have paid off, as 83% of the educators believed that they had successfully adapted their teaching style to suit an online environment. Among the more experienced lecturers, several comments indicate that the changes in teaching mode triggered by the pandemic prompted a more general update of the materials being taught, an improvement that was generally reflected in the student evaluations at the end of the term. In addition, a sense of pride for what had been achieved is present in many of the qualitative comments in the survey. Consider, for instance, the following reflection:

*‘*Students have been amazingly adaptive, patient and resilient. But I think we have all missed out from the lack of informal contact. In terms of my time— somehow preparation has taken much longer than usual and this term has often felt neverending and overwhelming. But I’m pleased and proud that we have managed to give students a pretty good experience in the circumstances.’

A majority of the university teaching community felt supported to make the transition to online teaching and learning: most of the academic staff felt supported by their department (75%), and by the university and its educational enhancement centre (at 50% and 58%, respectively) over the course of this process. The first port of call for many teachers were other colleagues and professional services staff within their own department, who by and large seem to have taken the leading role in helping each other deal with the shift to online teaching. The materials produced by the educational enhancement centre also appear to have influenced the way teachers approached the transition. Educators reporting higher levels of satisfaction with the support received from the centre were also more likely to employ student-centred teaching approaches in their online sessions—such as debates, student-led discussions, and shared documents in which students work together. Despite this, many others reported feeling overwhelmed given the massive amount of resources available. One common suggestion was *‘to have some pruning in the resources and advice—if there’s too much, you can’t see the forest for the trees’*. In this regard, several survey respondents emphasised the severe time pressure they were under, given the suddenness of the change in modes of teaching and the very unusual circumstances surrounding this change. Several blamed the school administration for not making speedier and clearer decisions on whether teaching would take place solely online. This may explain why a respondent stated that ‘*[their] department has done exceptionally well; the downside is that everyone is exhausted as workload has been overwhelming*’.

Despite all the issues that came with the transition to online teaching, most educators (54%) preferred to teach completely online or fully in-person rather than in hybrid classrooms. The most cited issues with hybrid classes involved technical difficulties, as well as concerns about being able to cater only to one side of the classroom while “losing” the other. But this did not mean that educators saw no value in retaining certain online elements while teaching on campus. For instance, a large number of respondents mentioned that holding office hours online proved more efficient and boosted student attendance. Several educators also highlighted the usefulness of lecture recordings—while keeping in-person sessions for discussion—and of integrating technology into traditional teaching approaches more broadly.

### Experimental analysis

The random assignment of the information nudge must imply that, on average, educators in both the treatment and control groups will feature similar background characteristics and teaching abilities. Consequently, they should display similar attitudes to online teaching in the absence of the nudge. This means that by comparing the attitudes of educators, in our sample, between the treatment and control groups, we can estimate the causal effect of the information nudge on educators’ perceptions about online teaching. To check if our randomisation was effective, we test for the balance of means of covariates between the control and treatment groups of educators in the sample. We do not find any significant differences in levels of these covariates, except for chance error^4^. Hence, we are confident that our randomisation strategy was effective (for details, see Table A.1. in Online Appendix).

**Fig. 1:**
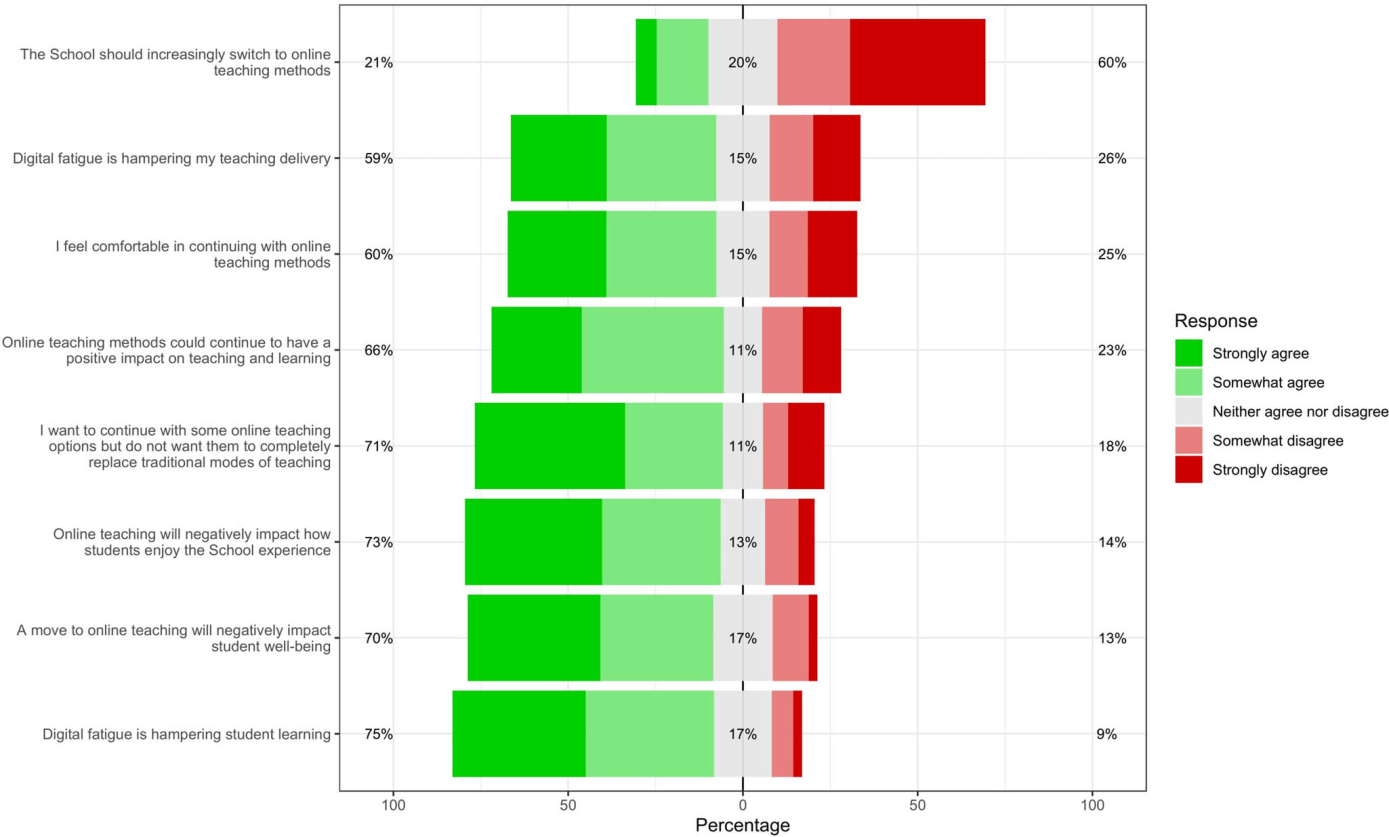
University educators’ perceptions regarding online teaching. Results shown follow a Likert scale. Percentages may not add up to 100 due to rounding.

Figure 1 shows participant agreement levels with the post-experimental measures described in the section “Experimental design”. Recall, after the information nudge was assigned randomly to educators, they were asked to indicate their agreement with a battery of 8 statements to indicate their new teaching beliefs for online teaching methods. Overall, these results show that respondents felt capable of teaching online. But, while most educators reported being comfortable with online teaching (60%), agreed with its potential to have positive impacts on learning (66%), and stated willingness to continue with some online teaching options, they disagreed with any further transition towards online modes of teaching (60%). In this regard, 71% of the educators felt that online teaching should not completely replace traditional (in-person) modes of teaching. Further, we find that, overall, student well-being and digital fatigue appeared as two important sources of concern. 70% of teachers believed online engagement methods negatively impacted student well-being as well as affected their overall experience at the institution (73%). In addition, three-quarters of educators subscribed to the notion that digital fatigue negatively impacted student learning, while about three-fifths reported it hampered their own teaching delivery.

Educators in the sample were largely in agreement on all these points. Any heterogeneity stemmed from more experienced teachers (and those who taught postgraduate courses), who appeared more comfortable with online teaching. They were also more likely to believe that online teaching methods could continue to have a positive impact on teaching. In contrast, we find that teachers who had undertaken a teaching certification course^5^ tended to have more negative views on the social aspect of online teaching, both in terms of student well-being and their enjoyment of the overall university experience.

Beyond these general views, nudging educators in the sample by positively reminding them of the benefits of online learning did not change their perceptions about online teaching. There are no significant differences in means between the treatment and control conditions. These results are shown in Table A.18. in the Online Appendix. There can be different possible explanations for our null findings, and we indicate two main ones. First, it is possible that our broad and neutral nudge did not sufficiently nudge educators to change their attitudes and beliefs. Second, we think that our nudge could also have been rendered ineffective in this context—particularly since educators had just completed a term of online teaching and were therefore likely to have retained some of their strong (negative) experiences. While we cannot confirm these hypotheses with our experimental data from this survey experience, qualitative evidence available in this survey indicated that the survey could have been perceived as patronising by educators. Consider the following comment:

"Now, this survey is probably well-intended, but it very much feels like another box-ticking exercise which ignores what teaching involves. It does not make me reflect on my teaching experience at all (*I don’t need a survey for that, thank you*) [emphasis added]”

Our thematic analysis of the open-ended survey questions further suggests that the mental states of educators could have shaped how they interacted with the survey and the nudge. In many instances, participants used open text boxes to voice negative attitudes, beliefs, or emotions about the survey, such as the excerpt above (for more details on analysis of open-ended comments in the survey, see section A in the Online Appendix).

## Conclusion

To summarise, we designed and administered an online survey experiment to 444 educators in a large social sciences university in the United Kingdom. Using this survey instrument, we evaluated their perceptions on the effectiveness of online teaching methods during the pandemic. Our findings suggest our information nudge, designed to inform educators about the benefits of online teaching, had limited effects in shaping the beliefs of educators in our sample about the efficacy of online teaching. Using this sample, we do not see any improvements in educators’ personal evaluations about this new mode of teaching. Overall, we find that most university teachers in the sample believe in the merits of online education, but do not support any further transition to this mode of teaching. We also find evidence that online teaching is believed by a majority of these educators to hamper students’ well-being and their overall university experience. Unlike previous findings in the literature (see section “Literature review”), we find that most university teachers in our sample felt supported by their department and the university in making this transition to online teaching.

While our research is timely, we are aware of some limitations. Our survey experiment was administered at an unprecedented time of heightened emotional and mental stress from COVID-19, which could drive many of our current findings. Further, as in other university settings, educators in our sample were adapting to a new form of teaching delivery during the time of the survey. As such, these mixed feelings that we find can be linked to the learning costs associated with maneuvering these new modes of teaching. Further, we are unable to include a representative sample of educators in our sample, even though we include representation from all departments in the university. While we believe that we represent a substantial part of the teaching community, our findings could be affected by self-selection bias, going both ways. For example, it could be that educators who were upset decided to take the survey to voice their dissatisfaction against online teaching methods, which would mean our findings are negatively biased and represent a lower-bound of the true sentiments. Or it could be that educators who were satisfied with online teaching voiced their opinions, in which case our findings are positively biased and represent an upper-bound of their true sentiments. As such, there is a need to externally validate our findings. Future research on nudging educators should also consider the effects of framing explicitly on educators’ beliefs and attitudes toward online modes of teaching.

We believe our research highlights the greater need for us to understand the sentiments, feelings, and behaviours of educators, as how they feel can trickle down to students and influence their learning experience. We also need to evaluate how these perceptions of online learning change over time, so we can effectively consider better pedagogical practices going forward. Our research has also highlighted the difference in needs and beliefs of educators to transition to online teaching tools—this variability should be better accounted for in policy design to increase the desired effectiveness of (educational) policy tools (for a general discussion, see Banerjee and Mitra, 2023). We are hopeful that future research will address these questions and build on our work. We end with a call for more experimental research to assess the role of *edunudges* in higher education settings.

## Supplementary information

Supplementary Materials

Figure A1

## Data Availability

The datasets generated during and analysed during the current study have been added to the Dataverse repository (for details, see Banerjee et al., 2023).

## Footnotes

1 Prior to the switch to online teaching prompted by the pandemic, the university had launched and/or was running 8 online certification courses as of March 2020. An estimated 20 academic faculty members and 12 administrative staff were involved in the organisation and delivery of these pre-pandemic online courses. Staff involved in such teaching did not receive any formal training in using online resources but were led through workshops organised by an external learning design team. This suggests that online modes of teaching introduced after the onset of the pandemic were in fact new to all faculty members.

2 When asked about hybrid teaching, over 75% of respondents agreed that hybrid teaching should be replaced either with fully on-campus or fully online teaching. Respondents’ open-ended responses point in the direction of two key problems: (1) educators found it difficult to engage two entirely separate audiences, and felt students joining online suffered as a result and (2) any technical issue would immediately derail the whole session.

3 This is a developmental centre within the university specialised in education and with a clear focus on academic staff development, curriculum enrichment, and digital innovation.

4 The gender of the educators, which is one out of 9 covariates tested, is unbalanced. We control for this covariate in our regression specification.

5 The Postgraduate Certificate in Higher Education is a necessary qualification for university lecturers to teach in the United Kingdom. Other junior members of staff, such as Graduate Teaching Assistants can also choose to complete it on a voluntary basis.
