# Supplementary material for: Nudge or not, university teachers have mixed feelings about online teaching
Source: Humanit Soc Sci Commun. 2023 May 12;10(1):232. doi: 10.1057/s41599-023-01691-1 (PMC10175908; doi:10.1057/s41599-023-01691-1)
Supplement: Supplementary file 1 — Supplementary Materials [file 41599_2023_1691_MOESM1_ESM.pdf]

Nudge or not, university teachers have mixed feelings about online teaching

Sanchayan Banerjee[*], Beatriz Jambrina-Canseco[†], Benjamin Brundu-Gonzalez[†], Claire Gordon[†], Jenni Carr[†]

April 7, 2023

[*]Correspondence to: S.Banerjee@vu.nl; Vrije Universiteit Amsterdam, The Netherlands
[†]London School of Economics and Political Science, United Kingdom

A Supplementary Materials

Methods for analysis of open-ended comments

What experiences, events, situations, or perceptions did participants had in mind when they completed the survey? What lived experience lurk behind the number?

To shed some light on these questions, we included seven several open questions in our survey. As open questions elicit unconstrained responses (?), we expected them to illuminate in more detail everyday perceptions and experiences associated to online teaching.

We collected a total 965 written answers. Of the 434 participants in this survey, 239 (55.07%) completed at least one-opened question and 40 (9.22%) completed all open-ended questions; aggregating all open-ended questions together, we found the overall response rate to open questions is 31.76%. These low response rates are routine with open-ended questions as they request more efforts from participants (?).

The open comments we collected varied in format and quality. The average written answer counted 16 words. We excluded from analysis those answers or parts of answers which meaning remained unclear after careful examination. This happened when answers only contained symbols, were too laconic, contained typos, language mistakes, or relied on words which meaning in context could not be decided.

To analyse this material, we opted for an inductive approach to qualitative coding (?). The coding process unfolded in four broad stages. First, we formatted the data for computer assisted coding in Nvivo (Saldana 2014). Second, we worked systematically through each written response to generate a great number of descriptive labels (?). Third, we reviewed our long collection of initial codes; and we fused some of them to guarantee their "external heterogeneity" and their "internal homogeneity" (?). Fourth, we organized our revised codes into broad themes and checked the internal homogeneity and external heterogeneity of each theme. The output of this analysis has been formalized as a codebook detailing the definitions of themes and codes along with examples taken from the material (available upon request).

Additional analysis

Balance Checks

Table A1: Teacher characteristics: Balance check

	(1) Control	(2) Treatment	(3) Mean diff.	(4) p-value
Gender (1 = Female, 2 = Male)	1.689	1.473	0.217	0.001
	(0.045)	(0.044)	(0.063)	
Age group	3.496	3.377	0.119	0.463
	(0.112)	(0.118)	(0.163)	
Foreign	0.471	0.589	-0.118	0.066
	(0.046)	(0.044)	(0.064)	
PGCert fellow	0.191	0.184	0.007	0.879
	(0.033)	(0.033)	(0.047)	
GTA or guest teacher	0.319	0.303	0.016	0.768
	(0.039)	(0.039)	(0.055)	
Teaching experience (in years)	12.768	11.937	0.831	0.534
	(0.993)	(0.891)	(1.334)	
Department	13.627	13.986	-0.359	0.660
	(0.568)	(0.587)	(0.817)	
Undergrad/postgrad teaching	1.979	1.937	0.042	0.671
	(0.069)	(0.072)	(0.099)	
Number of courses taught during LT	1.725	1.711	0.014	0.892
	(0.076)	(0.070)	(0.103)	
N	142	142		

Summary Statistics

Table A2: Teaching Roles of Educators

Teaching Role	Freq.	Percent	Cum.
Assistant Professor / Assistant Professorial Lecturer	52	12.44	12.44
Associate Professor / Associate Professorial Lecturer	70	16.75	29.19
Professor / Professorial Lecturer	82	19.62	100.00
Graduate Teaching Assistant	75	17.94	47.13
LSE Fellow (or equivalent) / Language Centre Teaching Staff	65	15.55	74.40
Guest Teacher	49	11.72	58.85
Other	25	5.98	80.38
Total	418	100.00	

Table A3: Professional Development Track of Educators

Career Track	Freq.	Percent	Cum.
Education Career Track	30	13.33	13.33
Other	16	7.11	20.44
Research and Teaching Track	179	79.56	100.00
Total	225	100.00	

Table A4: Primary Departmental Affiliations of Educators

Departmental Affiliation (Primary)	Freq.	Percent	Cum.
Data Science Institute	1	0.25	0.25
Department of Accounting	7	1.73	1.98
Department of Anthropology	16	3.95	5.93
Department of Economic History	19	4.69	10.62
Department of Economics	11	2.72	13.33
Department of Finance	8	1.98	15.31
Department of Gender Studies	3	0.74	16.05
Department of Geography and Environment	45	11.11	27.16
Department of Government	17	4.20	31.36
Department of Health Policy	19	4.69	36.05
Department of International Development	24	5.93	41.98
Department of International History	24	5.93	47.90
Department of International Relations	14	3.46	51.36
Department of Law	15	3.70	55.06
Department of Management	25	6.17	61.23
Department of Mathematics	23	5.68	66.91
Department of Media and Communications	11	2.72	69.63
Department of Methodology	14	3.46	73.09
Department of Philosophy, Logic and Scientific Method	9	2.22	75.31
Department of Psychological and Behavioural Science	8	1.98	77.28
Department of Social Policy	23	5.68	82.96
Department of Sociology	8	1.98	84.94
Department of Statistics	17	4.20	89.14
European Institute	16	3.95	93.09
Language Centre	26	6.42	99.51
Marshall Institute	1	0.25	99.75
School of Public Policy (formerly Institute of Public Affairs)	1	0.25	100.00
Total	405	100.00	

Table A5: Prior Teaching Experience of Educators

Prior University Teaching Experience	Freq.	Percent	Cum.
No, I have no prior university-level teaching experience	35	8.64	8.64
Yes, I have prior university-level teaching experience	370	91.36	100.00
Total	405	100.00	

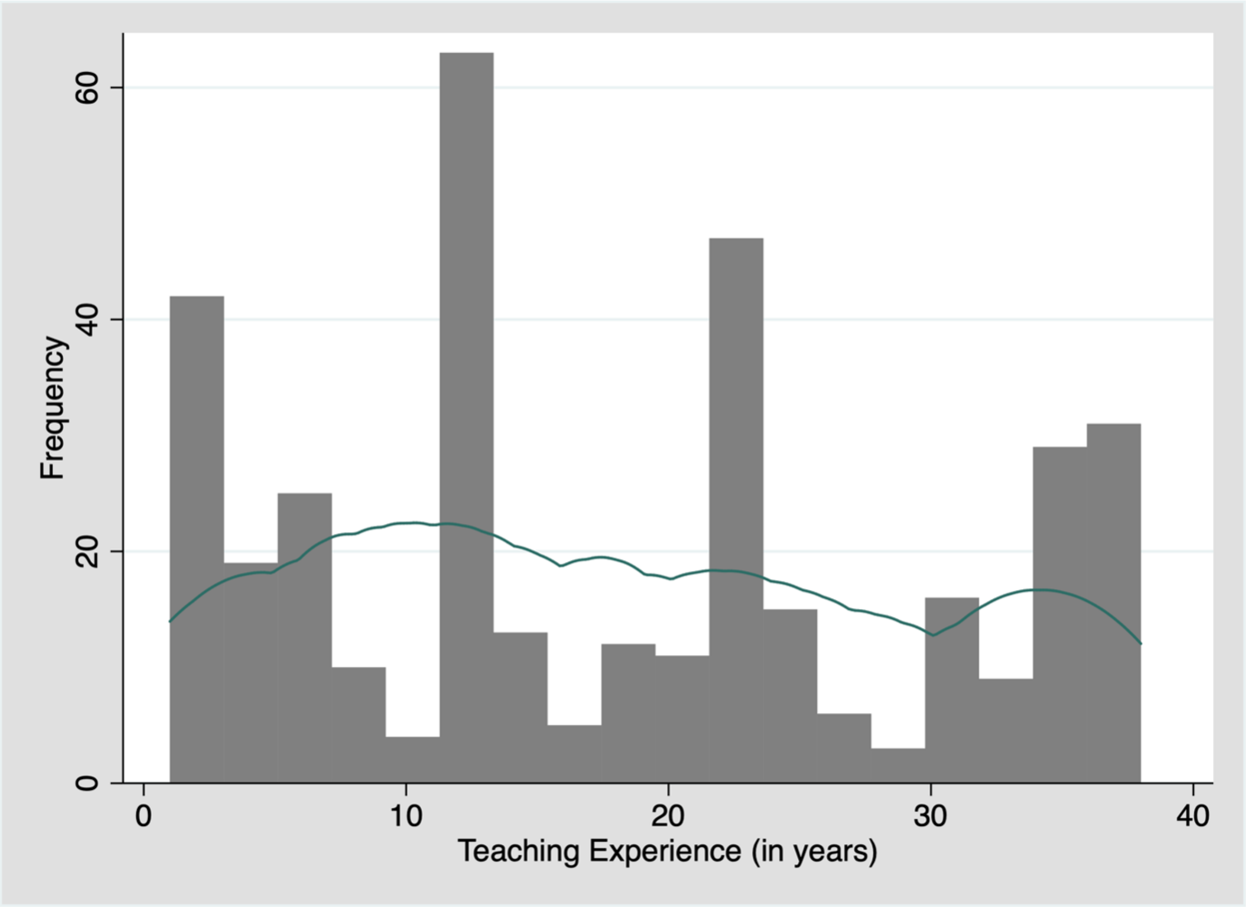

Figure A1: Prior years of university teaching experience of educators

Table A6: Teaching Load of Educators in Michaelmas Term

Michaelmas Term	Freq.	Percent	Cum.
1 course	170	43.04	43.04
2 courses	97	24.56	67.59
3 courses	29	7.34	74.94
4+ courses	23	5.82	80.76
I did not teach in this term	76	19.24	100.00
Total	395	100.00	

Table A7: Teaching Load of Educators in Lent Term

Lent Term	Freq.	Percent	Cum.
1 course	169	42.78	42.78
2 courses	134	33.92	76.71
3 courses	29	7.34	84.05
4+ courses	20	5.06	89.11
I did not teach in this term	43	10.89	100.00
Total	395	100.00	

Table A8: Dominant teaching mode of Educators

Dominant Teaching Mode	Freq.	Percent	Cum.
I taught on campus	17	6.75	6.75
I taught online	71	28.17	34.92
I taught using a hybrid format	44	17.46	98.81
I taught online and on campus	57	22.62	57.54
I taught online and using a hybrid format	21	8.33	65.87
I taught online, using a hybrid format and on campus	39	15.48	81.35
I taught using a hybrid format and on campus	3	1.19	100.00
Total	252	100.00	

Notes: Hybrid format includes teaching in a classroom where some of the students are online and some students are on campus

Table A9: Type of Class Teaching of Educators

Lecture/Class	Freq.	Percent	Cum.
Both lectures and classes/seminars	177	54.13	54.13
Class/seminars only	123	37.61	91.74
Lectures only	17	5.20	96.94
Other	10	3.06	100.00
Total	327	100.00	

Table A10: Level of Teaching of Educators

Level of teaching	Freq.	Percent	Cum.
Postgraduate	118	36.09	36.09
Undergraduate	100	30.58	66.67
Undergraduate and Postgraduate	109	33.33	100.00
Total	327	100.00	

Table A11: Additional Mentoring Responsibilities of Educators

Mentoring	Freq.	Percent	Cum.
No	129	39.45	39.45
Yes	198	60.55	100.00
Total	327	100.00	

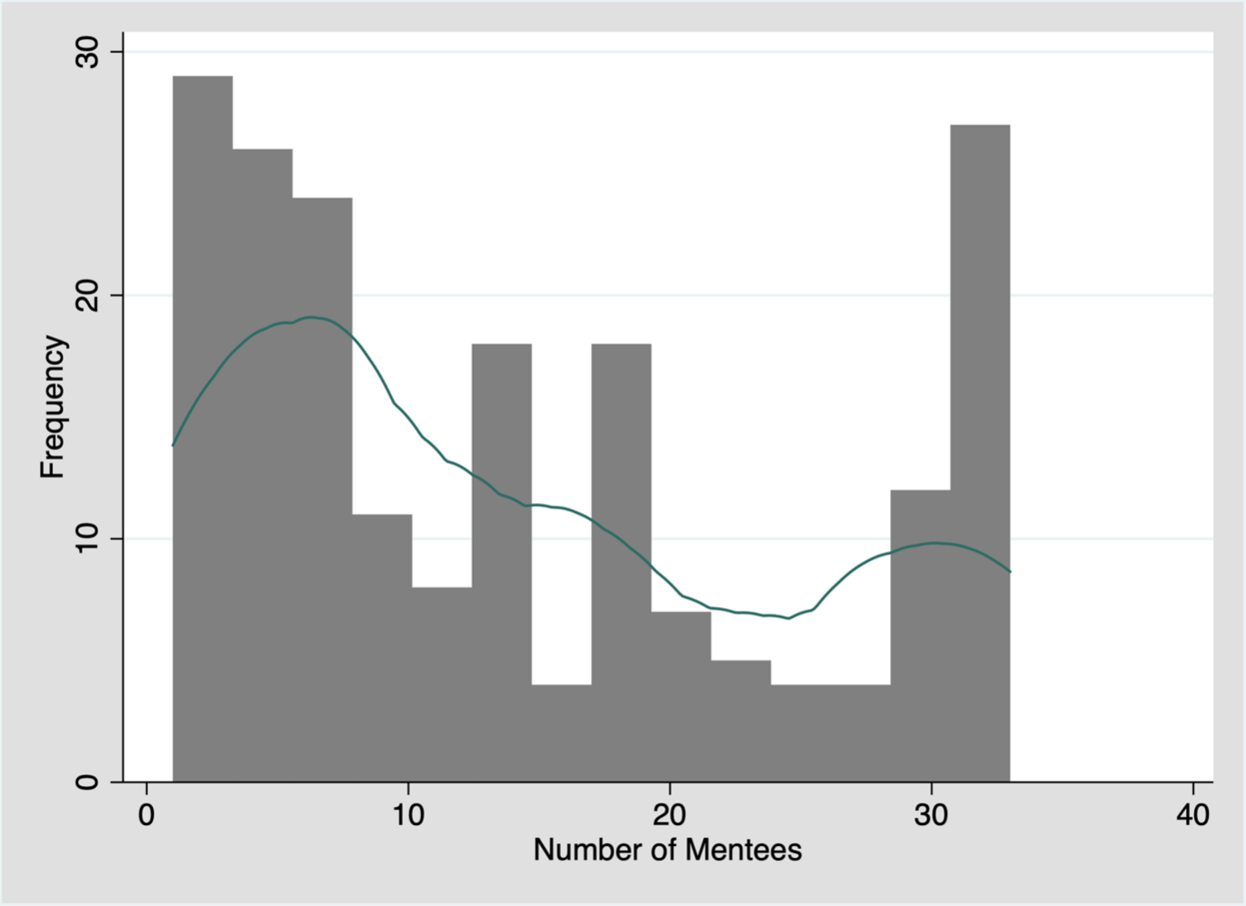

Figure A2: Number of mentees of educators

Table A12: University Teaching Accreditation of Educators

Fellow of the UK Higher Education Academy	Freq.	Percent	Cum.
I do not know about this Fellowship	101	35.82	35.82
No, I am not a Fellow of the UKHEA	128	45.39	81.21
Yes, I am a Full Fellow of the UKHEA	23	8.16	89.36
Yes, I am a Senior Fellow of the UKHEA	2	0.71	90.07
Yes, I am an Associate Fellow of the UKHEA	28	9.93	100.00
Total	282	100.00	

Table A13: Gender profile of Educators in Sample

Gender	Freq.	Percent	Cum.
Female	112	42.91	42.91
Male	146	55.94	98.85
Non-binary / third gender	3	1.15	100.00
Total	261	100.00	

Table A14: Age Profile of Sample

Age group	Freq.	Percent	Cum.
18 - 24	2	0.75	0.75
25 - 34	86	32.21	32.96
35 - 44	56	20.97	53.93
45 - 54	61	22.85	76.78
55 - 64	43	16.10	92.88
65 - 74	16	5.99	98.88
75 - 84	3	1.12	100.00
Total	267	100.00	

Table A15: Country of Residence of Educators at time of Survey

Country	Freq.	Percent	Cum.
Belgium	1	0.36	0.36
China	1	0.36	0.71
Czech Republic	1	0.36	1.07
France	1	0.36	1.43
Germany	2	0.71	2.14
Greece	1	0.36	2.50
Netherlands	1	0.36	2.86
Poland	1	0.36	3.21
Portugal	1	0.36	3.57
Russian Federation	1	0.36	3.93
Sweden	2	0.71	4.64
United Kingdom of Great Britain and Northern Ireland	263	93.93	98.57
United States of America	4	1.43	100.00
Total	280	100.00	

Table A16: Nationality of Educators in Sample

Nationality Status	Freq.	Percent	Cum.
Albania	1	0.41	0.41
Australia	1	0.41	0.82
Austria	1	0.41	1.22
Belgium	1	0.41	1.63
Brazil	3	1.22	2.86
Bulgaria	1	0.41	3.27
Canada	6	2.45	5.71
Chile	1	0.41	6.12
China	6	2.45	8.57
Colombia	2	0.82	9.39
Czech Republic	1	0.41	9.80
Finland	1	0.41	10.20
France	10	4.08	14.29
Germany	13	5.31	19.59
Greece	7	2.86	22.45
Hong Kong (S.A.R.)	1	0.41	22.86
Hungary	1	0.41	23.27
Ireland	6	2.45	25.71
Italy	18	7.35	33.06
Japan	3	1.22	34.29
Jordan	1	0.41	34.69
Liberia	1	0.41	35.10
Netherlands	6	2.45	37.55
Nigeria	1	0.41	37.96
Poland	2	0.82	38.78
Portugal	1	0.41	39.18
Romania	1	0.41	39.59
South Africa	1	0.41	40.00
Spain	6	2.45	42.45
Sweden	2	0.82	43.27
Switzerland	1	0.41	43.67
United Kingdom of Great Britain and Northern Ireland	115	46.94	90.61
United States of America	23	9.39	100.00
Total	245	100.00	

Table A17: What informs teaching opinions about student experiences?

Opinion Source	Freq.	Percent	Cum.
Informal feedback	36	12.04	12.04
Informal feedback ,Mid-term evaluations	7	2.34	14.38
Informal feedback ,Mid-term evaluations ,Other	1	0.33	14.72
Informal feedback,Other	14	4.68	19.40
Other	7	2.34	21.74
TQARO surveys	12	4.01	25.75
TQARO surveys,Informal feedback	143	47.83	73.58
TQARO surveys,Informal feedback,Informal Mid-term evaluations	48	16.05	89.63
TQARO surveys,Informal feedback,Informal Mid-term evaluations,Other	8	2.68	92.31
TQARO surveys,Informal feedback,Other	21	7.02	99.33
TQARO surveys,Informal Mid-term evaluations	2	0.67	100.00
Total	299	100.00	

Table A18: Average Treatment Effects of the Information Nudge

Variable	Student Fatigue	Teacher Fatigue	Comfort Online	Switch to Online	Positive Effects	Well-being	Negative Experience	Continue partially
Treatment dummy	-0.063	-0.103	0.227	-0.036	0.111	-0.177	0.087	-0.062
	(-0.49)	(-0.60)	(1.35)	(-0.22)	(0.70)	(-1.30)	(0.60)	(-0.37)
Controls (Non-zero)	0	0	0	0	0	0	0	0

t statistics in parentheses

* $p < 0.05$, ** $p < 0.01$, *** $p < 0.001$
