# Supplementary figures and images for: Nudge or not, university teachers have mixed feelings about online teaching
Source: Humanit Soc Sci Commun. 2023 May 12;10(1):232. doi: 10.1057/s41599-023-01691-1 (PMC10175908; doi:10.1057/s41599-023-01691-1)

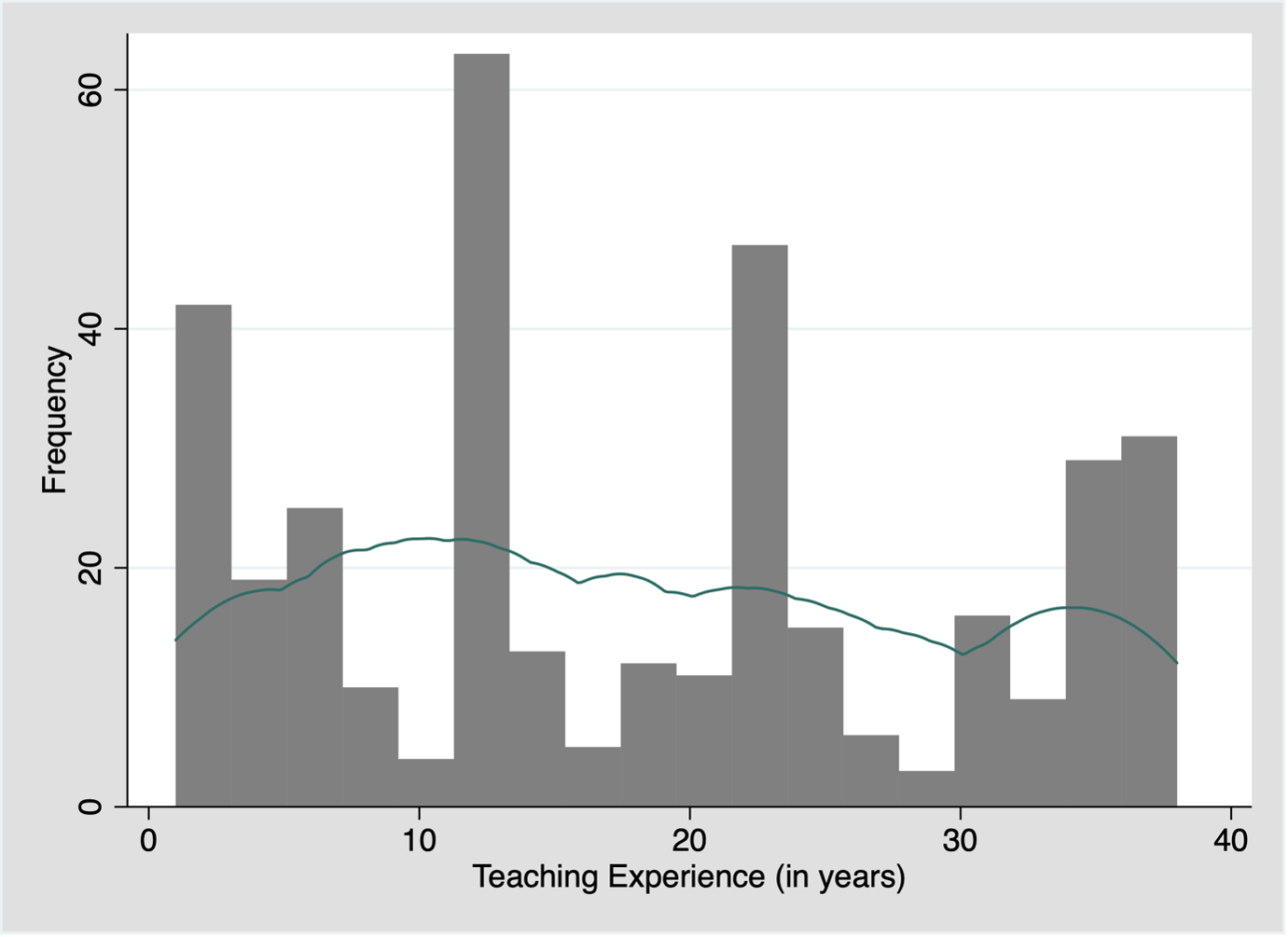

Supplement: Supplementary file 2 — Figure A1 [file 41599_2023_1691_MOESM2_ESM.png]
